# *Biosensors* in 2022

**DOI:** 10.3390/bios13030407

**Published:** 2023-03-21

**Authors:** Giovanna Marrazza

**Affiliations:** Department of Chemistry “Ugo Schiff”, University of Florence, 50019 Sesto Fiorentino (FI), Italy; giovanna.marrazza@unifi.it; Tel.: +39-055-457-3320

## 1. Introduction

Sixty years have passed since Clark and Lyons proposed the concept of using glucose enzyme electrodes to monitor the oxygen that is consumed during an enzyme-catalyzed reaction. In the decades that followed this proposal, advances in biotechnology, nanotechnology, and electronics made the realization of electrochemical and optical biosensors a hot research area. This Special Issue, “*Biosensors* in 2022”, is devoted to the most recent innovations and progresses in the area of biosensor technology, and consequently will stimulate researchers to identify and investigate their future applications in clinical, environmental, and food analyses. In total, 11 outstanding papers (including 6 research articles and 3 reviews, 1 communication and 1 perspective) were published in this Special Issue.

A biosensor has two components: a bioreceptor and a transducer. Researchers have continuously investigated both of these components, in order to improve the analytical performances and potential applications of these smart devices. The special characteristics of biosensors allow them to be used in screening and monitoring methods, especially when a continuous, real-time, in situ analysis is required.

In order to increase their stability, sensitivity, and selectivity, and to maintain the biological activity of the bioreceptor, a biosensor should consist of components with good biocompatibility. Therefore, numerous studies have been devoted to investigating the biocompatibility of these biosensors. Gelatin, a natural protein from animal tissue, has been applied in biosensor design as a matrix for bioreceptor immobilization, or as a biorecognition material for detecting various analytes. This is due to its excellent biocompatibility, biodegradability, biosafety, low cost, and sol–gel property. The aim of the perspective written by Tianyu Li et al. is to provide readers with an overview of the most recent important achievements of gelatin as an ideal component for biosensor realization [1].

The binding of drugs to DNA plays a key role in new drug discovery and is essential for designing enhanced drugs. Therefore, an explanation and visualization of the interaction mechanism between the DNA selective capture and the drug is of paramount importance. Uslu et al. conducted a molecular docking investigation as part of a biological assay, in order to predict the mode of the binding of the cinacalcet drug, which mimicked the action of calcium inside the DNA receptor [2]. 

It is known that neurotransmitters (NTs) play a central role in the modulation of various physiological brain functions, including behavior and cognition, cardiovascular, renal, and hormonal systems. Their real-time measurements within the living brain offer great benefits for the diagnosis and treatment of neurological disorders and diseases. Dopamine (DA) is a neurotransmitter belonging to the catecholamine family. It is involved in motor coordination, motivational behavior, and the regulation of cognitive processes such as attention and working memory. Xinyan Tracy Cui et al. presented an implantable glassy carbon multielectrode array (MEA) on a flexible substrate to obtain the integrated multichannel measurements of the DA concentrations and in vivo measurements [3]. 

However, for understanding the pathophysiology processes that are involved in brain disorders, due to the difficulty of carrying out in vivo studies, many researchers have used in vitro studies that utilize neuronal cell lines, because they are easily transfected, possess the ability to proliferate, and can be induced to differentiate into neuron-like cells, expressing neuronal biomarkers and presenting with axons and dendrites. The PC-12 cell line has been extensively assessed within neuroscience research for its shorter differentiation time and better neurogenic characteristics, as compared to other commercial immortal neuronal cell lines. Enache et al. reviewed the applications of different biosensors within PC-12 cell cultures and presented the modern approaches that are employed in neuronal network biosensing [4].

Studies on extracellular vesicles (EVs) are very helpful for medical screening and diagnostics. EVs are membrane-bound particles that contain proteins, nucleic acids, and other cargo that they have inherited from the mother cell, which secretes them into the extracellular space. The number of EVs that are isolated from the cell culture media depends on the cell state and is of considerable importance in clinical studies, including cell–cell signaling, disease modeling, and drug development studies, etc. Chernyshev and Skliar introduced the application of a quartz crystal microbalance as a biosensor for quantifying the EVs in a drop. This biosensing approach allowed for the number of EVs that were present in a sample to be determined in a short period of time and with a high precision [5].

The accurate and fast diagnosis of cancer disease plays a critical role in the timely and tailored treatment of patients. Therefore, the use of point-of-care (POC) tests is desirable for disease screening. Chiuan-Chian Chiou et al. developed an ultrafast polymerase chain reaction (PCR) platform using flow-through microchannel chips for the POC applications [6]. The template DNA for the establishment of this ultrafast PCR platform was genomic DNA from the leukemia cell line K562. The target amplicon was a 151-base-pair fragment within the actin gene, which is mostly used as a housekeeping gene for genetic testing. These results demonstrated that a simple chip design and fabrication are suitable for the development of commercial ultrafast PCR chips.

Biosensors are also employed to understand the mechanisms of cancer disease. Anchan et al. investigated the expression of metalloproteinases (MMPs) and other proteases in melanoma-conditioned media [7]. Melanoma is an aggressive skin cancer with a high tendency to metastasize to the blood–brain barrier via blood circulation. Here, brain endothelial cells form the first physical barrier that blood-borne cancer cells must breach. The obtained results by biosensors technology suggest that there is no direct evidence in the initial melanoma-mediated disruption of the brain endothelium, although melanoma cells do release some MMPs and proteases.

The development of sensitive and easy-to-use analytical methods for the detection of contaminants in situ are of paramount importance for environmental analysis and food safety.

Amine and Attaallah realized a sensitive and simple method for rapidly detecting cadmium ions (Cd^2+^) in water samples [8]. These authors presented two different approaches based on an enzymic membrane, using the easy and rapid immobilization method of horseradish peroxidase (HRP). Firstly, the inhibition of the horseradish peroxidase was performed using a colorimetric microplate reader. In addition, an electrochemical biosensor was designed by combining the enzymic membrane with screen-printed electrodes. In both formats, the biosensors were successfully employed to detect low cadmium ion concentrations.

Regarding food safety, there is an urgent need for sensitive, rapid and reliable analytical tools that are able to quickly detect allergen traces in food products. The presence of these allergens is often due to accidental contamination along the food supply chain (production, transformation, processing, and transport) and represents a high risk factor for allergic individuals, due to a potentially severe adverse reaction. Guardigli et al. summarized and discussed recent efforts in the field of food allergen analysis, using aptamer-based bioassays with luminescence detection [9].

Egg allergy is one of the most common food allergies, caused mainly by egg white proteins, with ovalbumin being the most abundant of these proteins. Mirasoli, Calabria et al. reported an origami paper-based device for detecting the ovalbumin in food samples, based on a competitive immunoassay with chemiluminescence detection [10]. The developed biosensor demonstrated a good assay specificity and accuracy, as compared with a commercial immunoassay kit. 

The applications of chemometric methods play a critical role in biosensor-based platforms and in improving their analytical characteristics. Chemometrics have been successfully applied in the development of electronic sensing platforms (ESPs) used for sensory analysis, in terms of an electronic nose (e-nose), electronic tongue (e-tongue), and electronic eye (e-eye). Altintas et al. reported the latest achievements and available solutions of the applications of these electronic sensing platforms (ESPs) for monitoring the chemical-based organoleptic attributes of tea and its products [11]. These authors examined the ESPs, which measure the tea’s aroma, taste, and color profiles, and input the data into mathematical classification algorithms, in order to discriminate between the different teas based on their price, geographical origins, harvest, fermentation, storage times, quality grades, and adulteration ratio. These promising results from using ESPs to maintain and improve tea quality demonstrate that similar electronic sensors can be used to predict and classify the sensory quality of other foods (juices, soft drinks, and milk, etc.), in order to satisfy the needs of consumers and the market.

## 2. Conclusions

This Special Issue on “*Biosensors* in 2022” provided an excellent platform for researchers to present their latest works and exchange ideas on the future directions of biosensors. The success of the articles submitted confirms the enormous potential of biosensors as new analytical tools within various fields. As researchers continue to explore this area, further progress will be achieved in the coming years. 
